# Spatial and Dietary Overlap Creates Potential for Competition between Red Snapper (*Lutjanus campechanus*) and Vermilion snapper (*Rhomboplites aurorubens*)

**DOI:** 10.1371/journal.pone.0144051

**Published:** 2015-12-02

**Authors:** William T. Davis, J. Marcus Drymon, Sean P. Powers

**Affiliations:** 1 Department of Ecology and Evolutionary Biology, Princeton University, Princeton, New Jersey, 08544, United States of America; 2 Department of Marine Sciences, University of South Alabama, LSCB Room 25, Mobile, Alabama, 36688, United States of America; 3 Center for Ecosystem Based Fishery Management, Dauphin Island Sea Lab, 101 Bienville Boulevard, Dauphin Island, Alabama, 36528, United States of America; North Carolina State University, UNITED STATES

## Abstract

Understanding the complex nature of direct and indirect species interactions is a critical precursor to successful resource management. In the northern Gulf of Mexico fisheries ecosystem, red snapper (*Lutjanus campechanus*) and vermilion snapper (*Rhomboplites aurorubens*) are two commercially harvested species within a larger reef fish complex. These two species share similar habitats and diets; however, little is known about how these species partition habitat and dietary resources. In this study we examined the extent of spatial and dietary overlap between red snapper and vermilion snapper, and experimentally compared their feeding behavior. Field data from multiple gear types demonstrates that red snapper and vermilion snapper frequently cohabited reefs in the northern Gulf of Mexico, and Pianka’s niche overlap indices suggest significantly overlapping diets. Experimental manipulations show that red snapper are the dominant forager of the two species, as red snapper foraging alone ate more shrimp per fish than vermilion snapper in both the single species (p = 0.003) and mixed species (p = 0.02) treatments. In addition, red snapper ate significantly more shrimp per fish in the mixed species treatment than in the single species treatment (p = 0.04). Vermilion snapper shrimp consumption per fish did not differ significantly between mixed and single species treatments. Cumulatively, our results suggest that spatial and dietary overlap could lead to competition between red and vermilion snapper in the study area; however, conclusively determining the existence of such competition would require further research.

## Introduction

Understanding species interactions can lead to a more holistic approach to the management of marine fisheries [1]. These interactions represent one of a suite of ecological processes that have the potential to influence the dynamics of fish stocks [2]. Some interactions, such as direct (*e*.*g*. predator-prey) trophic interactions, are conceptually straightforward and can be successfully incorporated into fishery management plans [3]. In contrast, indirect interactions, such as competition for a shared resource, are more subtle and thus harder to identify. Nonetheless, failure to account for these more nuanced interactions can lead to large-scale changes in ecosystem structure and function, and may push some ecosystems beyond their tipping point [4], alternative states from which the ecosystem may not recover.

Reef fish stocks are composed of multiple interacting species, many of which are slow growing, long-lived and late to mature [5]. The natural and artificial reefs of the Gulf of Mexico (GOM) support an ecologically diverse and economically important reef fish population. Two of the dominant species in this reef fish complex are red (*Lutjanus campechanus*) and vermilion (*Rhomboplites aurorubens*) snapper. Red snapper is the most economically important fish in the northern GOM, supporting a commercial fishery in excess of $10 million/year and a recreational fishery that has been estimated in value at $3 billion/year [6, 7]. The economic importance of vermilion snapper is similarly high with a commercial fishery in the GOM that has exceeded $8 million/year and a recreational fishery comparable to that of red snapper [7].

Red and vermilion snapper are associated with both high and low relief hard bottom habitat and thus have the potential for interaction. Examination of community structure using both camera [8, 9] and capture [10] gear demonstrates that these two species commonly co-occur, and that red snapper are numerically dominant. Previous analysis of each species separately [11, 12, 13] suggests there may be dietary overlap, as both species feed upon a similar variety of fish, decapods, and zooplankton. Competition between these two species has been previously suggested; Johnson et al. [12] speculated that the more dominant red snapper could force vermilion snapper to increase consumption of benthic tunicates, a normally undesirable food, in regions where the two species co-occur frequently.

Red and vermilion snapper have been well studied separately, and as components of a larger fish assemblage, yet studies directly comparing diet and behavior between these two species are lacking. To better understand the potential for indirect interactions, the degree of spatial and dietary overlap between these two species must first be established. To that end, the goals of this study were threefold. Our first objective was to establish the degree of spatial overlap between red and vermilion snapper using a combination of gear types. Our second objective was to examine the degree of dietary overlap between these two species, particularly in regions where they co-occur. Our third and final objective was to use controlled mesocosm feeding trials to determine if either species negatively affects the other’s ability to feed via exploitative or interference competition.

## Materials and Methods

### Ethics Statement

This study was conducted in accordance with the laws of the state of Alabama and under the IACUC protocols (IACUC Board Reference Number 11013) approved by the University of South Alabama. All sampling occurred in federal waters under permits granted by NOAA Fisheries to the authors. All efforts were made to reduce animal suffering during handling and tagging procedures.

### Field Sampling

**Fig 1:**
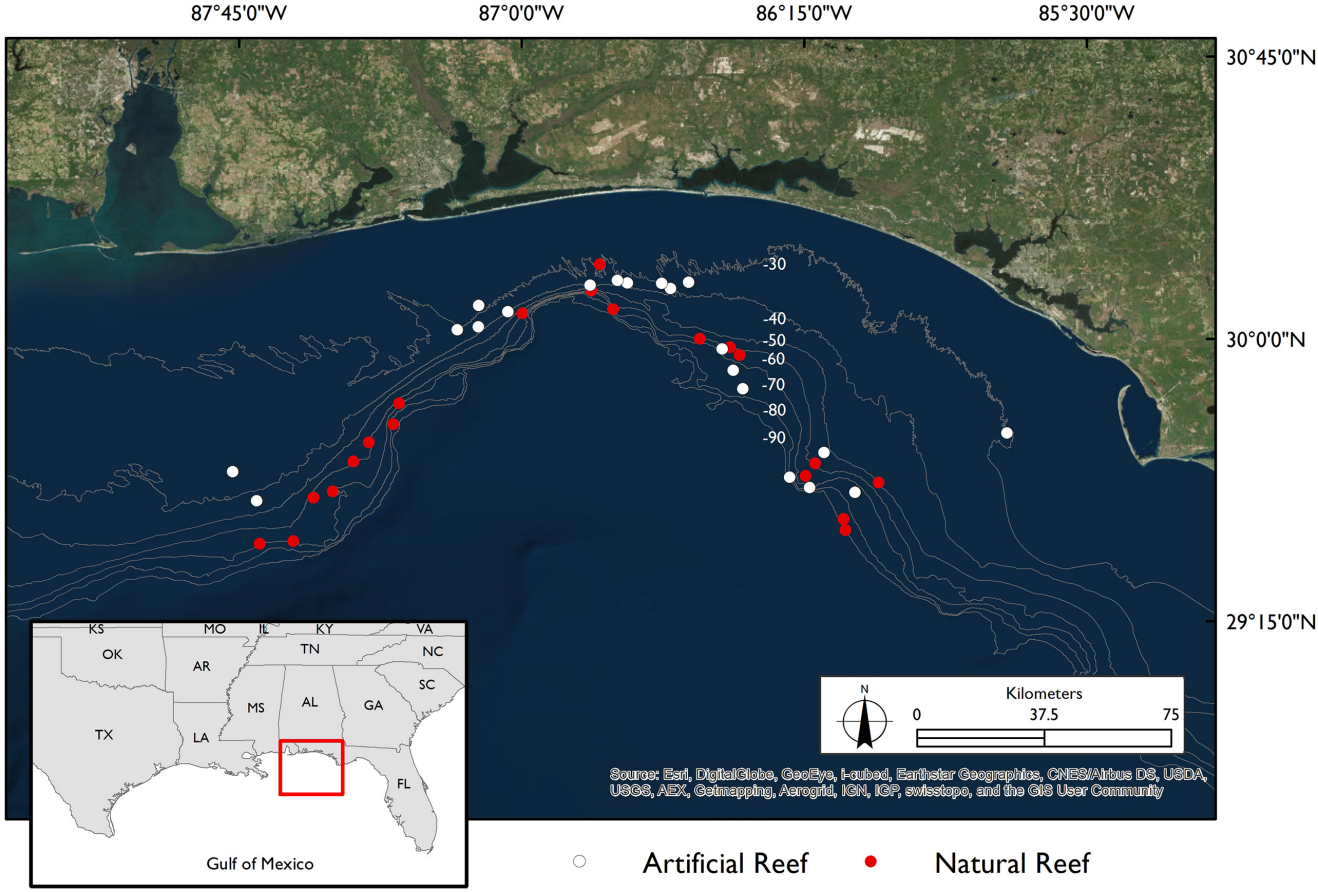
Map of area sampled. Forty reef locations, twenty artificial (white circles) and twenty natural (red circles) were sampled in the northcentral Gulf of Mexico off the coast of Alabama and Florida. White numbers on the map indicate bathymetry (in meters). Image generated using Esri ArcGIS v 10.3

Sampling was conducted during three eight-day research cruises from Spring 2011 to Spring 2012. Forty reef sites were sampled three times each, once during each cruise. All sampling was conducted during the day. The sampling sites were distributed evenly from Mobile Bay, AL to Saint Andrew’s Bay, FL. Half of the sampling sites were artificial reefs and half were natural reefs, with artificial and natural reefs distributed evenly across the sampling area (Fig 1). Three gear types were used to assess the community assemblage at each site. Remotely operated vehicle (ROV) was chosen as a non-extractive gear that minimizes the effects of size and species selectivity [14]; in addition, two capture gears, vertical longline and hook and line, were chosen as they represent the most common gear types used in the commercial and recreational fisheries, respectively. Specifically, before fishing each site, a Seabotix five-thruster LBV300-5 ROV [10] was deployed. The ROV recorded 4 minutes of stationary video from the sea floor facing the reef at two opposing locations 5 meters from the feature. The camera was pointed at a 45-degree angle with respect to the sea floor so that the reef and the adjacent water column would be included in the recording. Afterwards, the ROV was held 1 meter above the reef and a 360° panorama of the sea floor surrounding the feature was recorded for 1 minute. After recording ROV footage, all sites were fished using either vertical longline gear (3 standardized replicates)[10], or recreational hook and line gear (standardized to 30 minutes).

### Spatial Overlap

A combination of ROV and catch data was analyzed to establish spatial overlap, and determine potential habitat preferences (depth and/or reef type) between species. ROV videos were read in their entirety; differences in visibility between videos were negligible, and thus not accounted for in subsequent analyses. Presence/absence and MaxN [14] were determined for the following species: red snapper, vermilion snapper, grey snapper (*Lutjanus griseus*), lane snapper (*L*. *synagris*), scamp (*Mycteroperca phenax*), gag (*M*. *microlepis*), red grouper (*Epinephelus morio*), red porgy (*Pagrus pagrus*), and grey triggerfish (*Balistes capriscus*). As an initial approach for establishing spatial overlap, ROV and catch data were combined so that occurrence of any red snapper or vermilion snapper by any method indicated the presence of that species. Percentages of sites with neither species, only red snapper, only vermilion snapper, and both species present were calculated for each cruise and for all three cruises combined. To further examine habitat (depth and/or reef type) preferences, a permutational multivariate analysis of variance (PERMANOVA) was calculated on a square-root transformed, zero-adjusted Bray-Curtis similarity matrix [15] to establish if MaxN varied as a function of habitat using depth (20–40 m, 40–60 m, 60–80 m, 80–100 m), reef type (artificial or natural), and their interaction as factors. Pairwise tests were used to identify wherein significant differences identified by the PERMANOVA existed, and a similarity percentage test was used to further explore the species most responsible for driving any significant differences identified by the PERMANOVA.

### Dietary Overlap

Approximately half of the sites were randomly selected as tag-and-release sites, while at the other sites all fish were kept for stomach content analysis and age/sex assessment. Stomachs from all retained fish were removed and frozen shipboard. In the lab, stomachs were opened and contents were sieved through a 100 μm screen then identified to the lowest possible taxonomic level by examination under a dissection scope. For analysis, contents were categorized into 35 functional groups. Bait was excluded from the analysis. Each taxonomic group was then weighed to the nearest 0.01 gram. Percent composition by number (%N), percent frequency of occurrence (%O), percent composition by weight (%W), and percent index of relative importance (%IRI) were calculated according to the following equations [16]:

$$\%N_{\text{i}}=\frac{number\;of\;prey\;type\;i}{sum\;total\;of\;all\;identifiable\;prey\;from\;all\;categories}\;\times \;100$$ (1)

$$\%O_{i}=\;\frac{number\;of\;stomachs\;containing\;prey\;category\;i}{number\;of\;stomachs\;with\;identifiable\;prey\;present}\;\times \;100$$ (2)

$$\%W_{i}=\;\frac{weight\;of\;prey\;type\;i}{weight\;of\;total\;identiable\;stomach\;contents}\;\times \;100$$ (3)

$$IRI_{i}=\left(\%N_{i}\;\times \;\%W_{i}\right)\;\times \;\%O_{i}$$ (4)

$$\%IRI_{i}=\;\frac{IRI\;for\;each\;prey\;category\;i}{sum\;of\;all\;IRI\;values}\;\times \;100$$ (5)

Dietary characterization and overlap for red and vermilion snapper were investigated using multivariate and null model approaches. First, a PERMANOVA was used to examine whether stomach contents (measured by %W) in red and vermilion snapper varied with season (spring, summer), reef type (natural, artificial), depth (20–60 m, 61–100 m), and cohabitation (presence/absence of red or vermilion snapper, depending). Main effects and all first order interactions were calculated separately for each species based on a square-root transformed, zero-adjusted Bray-Curtis similarity matrix [15]. Similarity percentage (SIMPER) tests were used to further explore the prey categories most responsible for driving any differences observed as a function of season, reef type, depth, cohabitation, or their first order interactions. Second, Pianka’s niche overlap [17] was calculated and compared to a null expectation (RA3 algorithm, 1000 repetitions) using the R package EcoSimR [18].

### Mesocosm Experiment

Red and vermilion snapper were collected for use in mesocosm experiments during five trips in June and July 2013 via hook and line fishing from artificial reef sites located 11–19 km south of Dauphin Island, AL. The target size class was 250–350 mm FL. The fish were collected over shallow depths (19–28 meters) to avoid barotrauma and were handled as little as possible after catch. Fish were transported back to Dauphin Island Sea Lab in a 170.3 L (45 gal.) flow-through livewell. After capture and return to the lab, fish were held in holding tanks and allowed a two-week acclimation period before being used in trials. Fish were observed closely during acclimation to ensure that they were feeding before being used in trials. All fish were housed in outdoor, shaded tanks with a recirculating pump and filter system. The tanks were 2.44 m (8 ft) in diameter and 1.07 m (3.5 ft) in depth, (filled to approximately 1 m in depth), translating to a volume of roughly 4100 liters. Temperature, salinity, and pH levels were monitored daily. Fish were fed a mixed diet of squid (*Loligo sp*.), penaeid shrimp (*Penaeus aztecus*), and mackerel (*Scomber scombrus*) until satiation every 48 hours. Experimental tanks were identical to holding tanks, with the following exceptions. Experimental tanks contained two hollow concrete cinder blocks to provide shelter, and had sand covering the bottom of the tank. The availability of these small shelters seemed to allow the fish to acclimate more quickly to the experimental tanks. The sand bottom allowed shrimp to attempt to hide by partially burrowing as they would in a natural environment. Both the holding and experimental tanks were part of the same recirculating system.

The experiment employed a substitutive design and consisted of four treatments: 1) 0 red snapper and 0 vermilion snapper (control), 2) 6 red snapper and 0 vermilion snapper, 3) 0 red snapper and 6 vermilion snapper, and 4) 3 red snapper and 3 vermilion snapper. For all treatments 15 brown shrimp (*Farfantepenaeus aztecus*) 90–100 mm TL were introduced to the experimental tank, and the first 30 minutes of feeding after introduction of prey were recorded with a pair of GoPro Hero2 cameras in the tank (S1 Fig). Brown shrimp were chosen because they are common to the diet of both species, easily visible during video analysis, and readily available. Following the trials, the video was viewed and consumption of shrimp and failed predation attempts were enumerated for each species. Consumption of the shrimp prey by red or vermilion snapper defined a successful predation attempt. Conversely, a failed predation attempt was defined as movement of the fish towards the shrimp prey with an unsuccessful feeding strike (S1 File). The time of these predation events was also noted so that feeding rates might be examined. In order to examine feeding rates, time elapsed and species were examined as factors influencing cumulative shrimp consumption. Cumulative shrimp consumption was square transformed to achieve linearity.

The fish were starved for 48 hours before beginning trials to ensure a standardized hunger level. Fish were randomly selected for trials from pools of 8–14 vermilion snapper and 12–18 red snapper. Fish being used in trials were transferred from holding tanks to experimental tanks 24 hours prior to commencing the trial to allow for acclimation and recovery from any stress resulting from transfer. Stress relating to transfer was assumed to be negligible, as the transfer process was very quick and holding and experimental tanks were less than 3 meters apart. No fish died following transfer.

## Results

### Spatial Overlap

**Fig 2:**
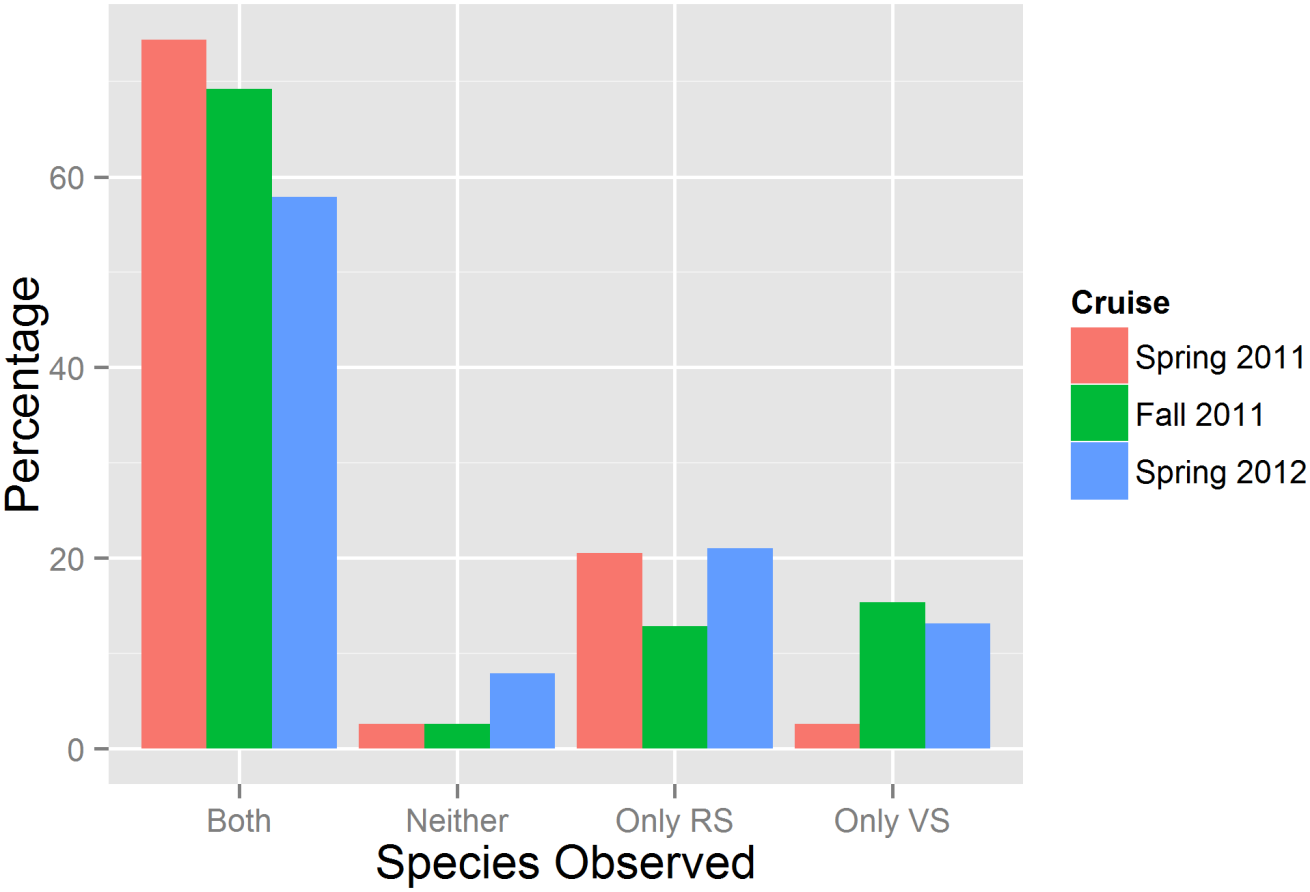
Red and vermilion snapper spatial overlap. Percentage of sites where both red snapper (RS) and vermilion snapper (VS) were observed, only red snapper were observed, only vermilion snapper were observed, and neither species was observed during three research cruises in Spring 2011, Fall 2011 and Spring 2012.

**Fig 3:**
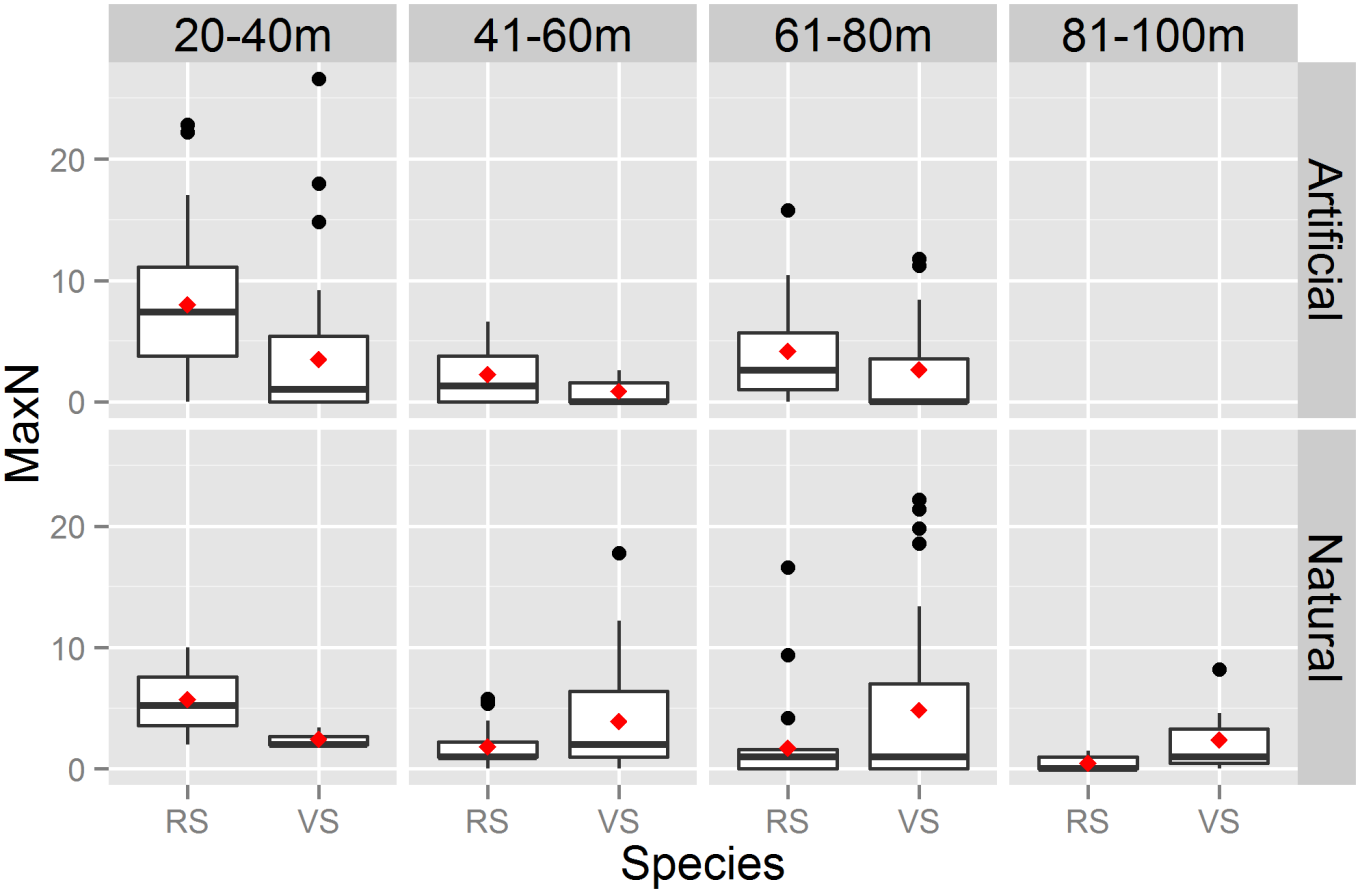
Red and vermilion snapper habitat preference. MaxN of red snapper (RS) and vermilion snapper (VS) measured via ROV for all three research cruises combined. The boxes represent the interquartile ranges (IQR); the horizontal black line is the median; the red diamond indicates the mean. The whiskers indicate the most extreme value within 1.5 × IQR. All black dots are outliers (further than 1.5 × IQR from the median).

ROV video and catch data analysis showed that red and vermilion snapper frequently cohabited reefs across all three research cruises. The two species overlapped at 74%, 69%, and 58% of sites sampled during the first, second, and third cruises, respectively (Fig 2). According to ROV data, red and vermilion snapper were the two most abundant species for all three cruises. Mean red snapper MaxN for all three cruises combined was 4.58, and mean vermilion snapper MaxN was 3.66, more than double the mean MaxN for any other species encountered. Results from the PERMANOVA revealed habitat related differences in community assemblage as a function of both depth (pseudo F_3,112_ = 3.15, p< 0.01) and reef type (pseudo F_1,112_ = 2.37, p< 0.05). Furthermore, a non significant interaction (pseudo F_2,112_ = 1.10, p = 0.372) between depth and habitat suggests that these factors are independent, such that further exploration is warranted. With respect to depth, pairwise tests indicated that the largest differences were between the fish assemblages at 20-40m compared to 41-60m (p<0.01) and 61-80m (p = 0.01). Similarity percentage tests demonstrated that these community assemblage differences were driven primarily by red and vermilion snapper. Red snapper were more than twice as abundant (mean MaxN = 2.56) in waters 20-40m compared to deeper water (mean Max N = 1.18 in 40–60, and 60–80). Conversely, vermilion snapper MaxN gradually increased as depth increased. Examining reef type across all sampled depths, red and vermilion snapper were again the two species most responsible for the community assemblage differences identified, with red snapper MaxN more than twice as high (2.12) on artificial reefs compared to natural reefs (1.02), and vermilion snapper MaxN higher on natural reefs (1.51) relative to artificial reefs (1.09) (Fig 3).

### Stomach Contents and Dietary Overlap

Stomachs from 154 vermilion snapper (199–496 mm FL, mean = 338 mm FL) were sampled over 49 unique site/days from 25 stations, and stomachs from 216 red snapper (308-727mm FL, mean = 501mm FL) were sampled over 38 unique site/days from 20 stations. Of those, 92 of the vermilion snapper stomachs and 133 of the red snapper stomachs contained identifiable material (chyme). Unidentifiable material comprised 80.4% and 76.1% of stomach content mass for vermilion and red snapper, respectively. Unidentifiable material was excluded from all calculations presented hereafter. Among the identifiable contents, 46 unique prey taxa were identified (S1 Table). To make valid comparisons between the two species of interest, and for comparison with previously published dietary studies on red snapper [11, 13, 19, 20] and vermilion snapper [12], consumed prey were condensed into 28 broader categories for analysis. Amphipods comprised 22.1% and 47.3% of vermilion snapper stomach contents by %W and %IRI, respectively, and fish comprised 36.2% and 56.0% of red snapper stomach contents by %W and %IRI, respectively (Table 1).

**Table 1:** Stomach contents of red snapper and vermilion snapper collected in the northern Gulf of Mexico. Only categories with %IRI > 1 for either red snapper or vermilion snapper are shown. %N = percent by number, %W = percent by weight without unidentified stomach contents, %O = frequency of occurrence, and %IRI = index of relative importance, expressed as a %. All values are means, with ±SE shown for %N and %W.

	%N		%W		%O		%IRI	
Prey Category	RS	VS	RS	VS	RS	VS	RS	VS
**Amphipod**	11.12 ± 2.44	25.56 ± 3.54	8.19 ± 2.21	22.12 ± 3.82	16.54	48.91	6.65	47.26
**Copepod**	5.86 ± 1.77	10.75 ± 2.57	0.17 ± 0.12	5.56 ± 2.15	9.77	23.91	1.23	7.90
**Crab**	9.56 ± 2.13	11.99 ± 2.94	11.41 ± 2.49	12.78 ± 3.16	21.80	28.26	9.52	7.53
**Fish**	27.61 ± 3.50	10.93 ± 2.79	36.19 ± 4.02	16.66 ± 3.74	42.11	25.00	55.96	13.97
**Gastropod**	16.72 ± 2.85	4.35 ± 1.74	16.01 ± 2.90	3.12 ± 1.65	26.32	14.13	17.94	2.14
**Krill**	0.28 ± 0.28	3.67 ± 1.57	0.31 ± 0.31	2.68 ± 1.40	0.75	11.96	0.01	1.54
**Mantis Shrimp**	4.46 ± 1.56	6.90 ± 2.01	5.69 ± 1.85	12.00 ± 3.06	12.03	23.91	2.54	9.16
**Ostracod**	1.83 ± 0.99	3.60 ± 1.51	0.81 ± 0.79	1.13 ± 0.72	4.51	14.13	0.25	1.36
**Shrimp**	4.81 ± 1.50	6.78 ± 2.09	5.27 ± 1.77	7.14 ± 2.29	12.78	18.48	2.69	5.21
**Tunicate**	3.74 ± 1.32	4.78 ± 2.04	3.31 ± 1.35	6.67 ± 2.51	8.27	6.52	1.21	1.51

The diets of red and vermilion snapper varied as a function of habitat and season, respectively. For red snapper, diet varied between artificial and natural reefs (PERMANOVA pseudo-F_1,116_ = 7.20, p<0.01, Table 2). Red snapper diets at artificial and natural reefs were highly dissimilar (average Bray-Curtis dissimilarity = 77.41%), largely the result of habitat-related differences in fish consumption. By weight, fish contributed less to the diet of red snapper at artificial habitats (%W SIMPER metric = 3.01) compared to natural habitats (%W SIMPER metric = 7.24). Diet of red snapper on artificial reefs was further characterized by low contribution from crab (1.34 compared to 1.73%W SIMPER metric) and higher contribution from gastropods (2.40 compared to 0.05%W SIMPER metric). Vermilion snapper diet was relatively consistent across habitat, yet varied with season (PERMANOVA pseudo-F_1,78_ = 5.63, p<0.01). Vermilion snapper diets in spring and summer were highly dissimilar (average Bray-Curtis dissimilarity = 90.81%), driven primarily by seasonal differences in amphipods in vermilion snapper diets. By weight, amphipods were much more prevalent during the spring (%W SIMPER metric = 4.10) compared to the summer (%W SIMPER metric = 0.31). Vermilion snapper diet in the spring was further characterized by lower contribution from crab (0.92 compared to 2.91%W SIMPER metric) and higher contribution from fish (2.40 compared to 0.97%W SIMPER metric).

**Table 2:** Stomach contents of red snapper and vermilion snapper divided by reef type. Only categories with %IRI > 1 for either red snapper or vermilion snapper are shown. %W = mean percent by weight without unidentified stomach contents, ±SE indicates the standard error. %IRI = index of relative importance, expressed as a %.

	Artificial Reefs				Natural Reefs			
	%W		%IRI		%W		%IRI	
Prey Category	RS	VS	RS	VS	RS	VS	RS	VS
Amphipod	10.30 ± 2.74	20.31 ± 7.21	10.06	51.23	0.00 ± 0.00	22.87 ± 4.53	0.24	46.61
Copepod	0.21 ± 0.16	10.66 ± 5.76	1.76	5.50	0.00 ± 0.00	3.45 ± 1.86	0.11	1.49
Crab	10.91 ± 2.75	18.16 ± 6.55	9.38	19.38	13.36 ± 5.84	10.56 ± 3.55	6.60	4.60
Fish	27.70 ± 4.19	4.01 ± 3.84	38.19	2.75	69.18 ± 8.30	21.87 ± 4.90	89.50	20.66
Gastropod	20.12 ± 3.53	5.90 ± 4.15	29.68	5.83	0.07 ± 0.07	1.97 ± 1.59	0.06	1.20
Krill	0.40 ± .40	2.56 ± 2.56	0.02	0.39	0.00 ± .00	2.73 ± 1.68	0.00	2.14
Mantis Shrimp	5.13 ± 1.90	10.20 ± 5.36	2.41	6.47	7.85 ± 5.32	12.74 ± 3.74	1.81	10.47
Ostracod	1.02 ± 0.99	0.00 ± 0.00	0.43	0.39	0.00 ± 0.00	1.59 ± 1.01	0.00	1.82
Shrimp	6.49 ± 2.21	5.54 ± 4.14	3.13	3.04	0.54 ± 0.32	7.80 ± 2.77	0.86	6.22
Tunicate	4.16 ± 1.70	7.53 ± 5.22	1.83	0.97	0.00 ± 0.00	6.32 ± 2.85	0.05	1.73

Cohabitation, i.e. the presence of either red or vermilion snapper, was not a significant factor influencing the dietary composition of either of these two species; however, the diets of both species were highly dissimilar in sympatric vs allopatric conditions (Table 3). The interactions between cohabitation and habitat type (reef type and depth) were not significant, so the effects of cohabitation were independent of variations in habitat. The contribution of fish (%W) was similar in the stomachs of red snapper sampled without vermilion snapper (%W SIMPER metric = 4.03) compared to stations where both red and vermilion snapper were sampled (%W SIMPER metric = 3.63). Red snapper sampled without vermilion snapper were characterized by lower contributions (%W) of gastropods (1.73 vs 2.23%W SIMPER metric) and crabs (1.06 vs 2.01%W SIMPER metric) than at sites where the two species co-occurred. Vermilion snapper sampled at stations with both red and vermilion snapper had higher prevalence of amphipods (%W) in their stomachs (%W SIMPER metric = 3.35) compared to locations with only vermilion snapper (%W SIMPER metric = 1.55).

**Table 3:** Stomach contents of red snapper and vermilion snapper from cohabited (sympatric) and single-species (allopatric) sites. Only categories with %IRI > 1 for either red snapper or vermilion snapper are shown. %W = mean percent by weight without unidentified stomach contents, ±SE indicates the standard error. %IRI = index of relative importance, expressed as a %.

	Allopatric Conditions				Sympatric Conditions			
	%W		%IRI		%W		%IRI	
Prey Category	RS	VS	RS	VS	RS	VS	RS	VS
Amphipod	8.57 ± 2.92	9.91 ± 4.67	6.93	20.88	7.59 ± 3.38	28.33 ± 5.08	5.74	59.31
Copepod	0.27 ± 0.20	5.10 ± 3.55	3.24	9.47	0.00 ± 0.00	5.80 ± 2.71	0.00	6.40
Crab	8.37 ± 2.73	8.46 ± 4.14	5.46	4.52	16.25 ± 4.72	14.98 ± 4.27	17.01	8.47
Fish	37.92 ± 5.23	22.37 ± 7.00	60.57	23.14	33.45 ± 6.30	13.75 ± 4.37	45.81	9.04
Gastropod	13.47 ± 3.24	5.46 ± 3.60	13.86	4.22	20.05 ± 5.46	1.93 ± 1.70	23.65	1.15
Krill	.51 ± .51	7.81 ± 4.02	0.02	9.36	0.00 ± 0.00	0.07 ± 0.05	0.00	0.02
Mantis Shrimp	6.26 ± 2.46	16.47 ± 6.18	2.82	16.72	4.78 ± 2.80	9.72 ± 3.39	1.99	5.42
Ostracod	1.32 ± 1.28	0.25 ± 0.20	0.52	1.12	0.00 ± 0.00	1.57 ± 1.08	0.01	1.35
Shrimp	6.70 ± 2.72	11.74 ± 5.27	2.70	7.52	3.00 ± 1.52	4.81 ± 2.17	2.27	3.58
Tunicate	5.29 ± 2.18	6.28 ± 4.36	1.95	0.54	0.15 ± 0.15	6.87 ± 3.10	0.36	2.04

Pianka’s overlap indices indicate significant overlap between red and vermilion snapper stomach contents (Table 4). Overlap with respect to %W was 0.74 (p = 0.002) and overlap with respect to %N was 0.65 (p = 0.01). Overlap with respect to %IRI was 0.40 (p = 0.025). While still significant, overlap with respect to %IRI was lower due to large differences between red and vermilion snapper stomach contents in the categories “fish” and “amphipods”. Vermilion snapper had a much larger %IRI for amphipods, while red snapper had a much larger %IRI for fish; however, each category ranks in the top four categories by %IRI for both species.

**Table 4:** Pianka's niche overlap indices. Values are given for overlap indices for stomach contents collected from all sites, cohabited sites (sympatric), and single-species sites (allopatric).

	All Sites		Sympatric Conditions		Allopatric Conditions	
	Pianka's Index	p-value	Pianka's Index	p-value	Pianka's Index	p-value
%IRI	0.40469	0.025	0.28163	0.08	0.70298	0.001
%N	0.64887	0.01	0.49207	0.07	0.76185	0.001
%W	0.74122	0.002	0.61908	0.024	0.85092	0.001

Pianka’s overlap indices were also calculated for the subsets of red and vermilion snapper stomachs collected at sites the two species cohabited (sympatric conditions) and sites where only one species was observed (allopatric conditions). At single species sites (allopatric conditions) overlap was very high with respect to %W (index = 0.85, p = 0.001), %N (index = 0.76, p = 0.001), and %IRI (index = 0.70, p = 0.001). At cohabited sites (sympatric conditions) overlap was lower than at single species sites and was not significant with respect to %N (index = 0.49, p = 0.07) and %IRI (index = 0.28, p = 0.08). Overlap with respect to %W was significant at cohabited sites (index = 0.62, p = 0.024) but was much lower than at single species sites (Table 4).

### Mesocosm Experiment

Eight replicates of the red snapper and vermilion snapper single species treatments were completed; however, only 6 replicates of the mixed species treatments were completed. Red and vermilion snapper used in trials were of similar sizes: mean red snapper length was 273 (±8.57) mm FL and mean vermilion snapper length was 285 (±3.79) mm FL. Red snapper mean mass was 0.36 (±0.035) kg and vermilion snapper mean mass was 0.33 (±0.018) kg.

**Fig 4:**
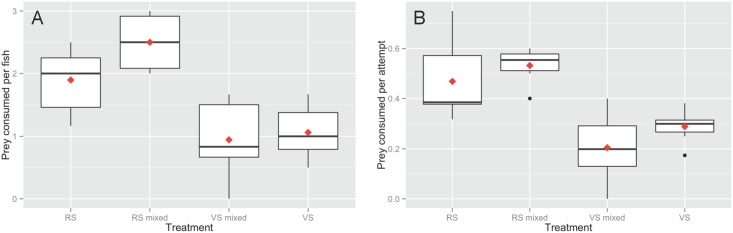
Red and vermilion snapper experimental prey consumption. (A) Shrimp consumed per fish in each of four treatment groups. Data shown represent consumption by: red snapper alone (RS), red snapper in the mixed treatment (RS mixed), vermilion snapper (VS) in the mixed treatment (VS mixed), and VS alone. (B) Shrimp consumed per attempt across the same four treatments. The boxes represent the interquartile ranges (IQR); the horizontal black line is the median; the red diamond indicates the mean. The whiskers indicate the most extreme value within 1.5 × IQR. All black dots are outliers (further than 1.5 × IQR from the median).

Red snapper from the single species treatments ate significantly more shrimp per fish than vermilion snapper in both the single species (p = 0.003) and mixed species (p = 0.02) treatments. In the single species treatments red snapper consumed an average of 0.83 more shrimp per fish than vermilion snapper. In the mixed species treatment red snapper consumed an average of 1.56 more shrimp per fish than vermilion snapper. Red snapper ate significantly more shrimp per fish in the mixed species treatment than in the single species treatment (p = 0.04). Vermilion snapper shrimp consumption per fish did not differ significantly between mixed and single species treatments (Fig 4a). Linear regression revealed that shrimp consumption per fish was highly correlated with predation success rate across all treatments (Fig 4b, R^2^ = 0.56; df = 1, 26; F = 32.55; p<0.001).

**Fig 5:**
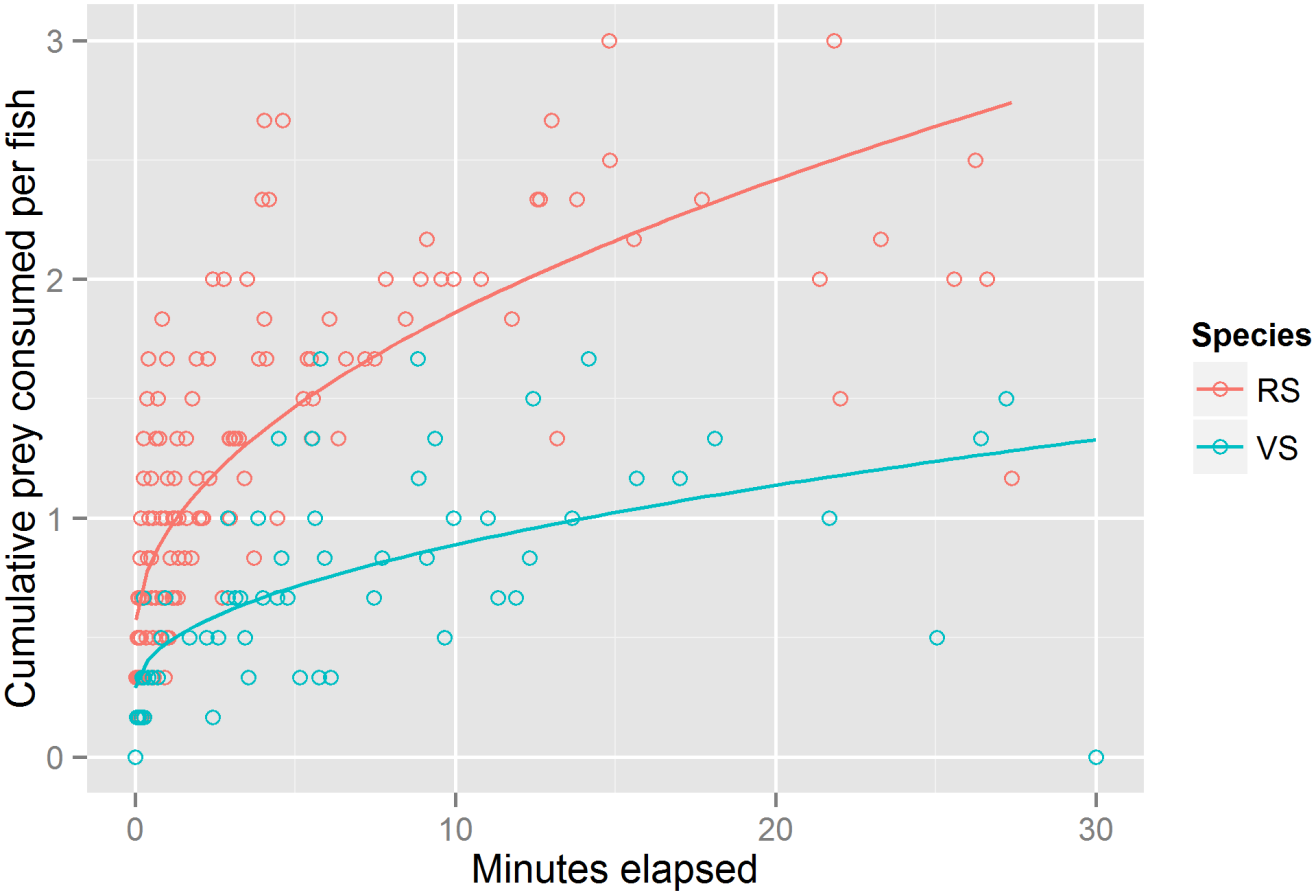
Red and vermilion snapper experimental feeding rates. Cumulative shrimp consumed vs. minutes elapsed during mesocosm trials. Best-fit lines are added for clarity.

Red snapper fed at a higher initial rate than vermilion snapper in both the single species and mixed species treatments (Fig 5). The overall model for cumulative shrimp consumption with elapsed time and species as factors was significant (R^2^ = 0.50; df = 3, 196; F = 65.7; p<0.001). The interaction term for time elapsed and species was significant, indicating that time elapsed better predicted cumulative shrimp consumed for red snapper than for vermilion snapper (F = 33.7, df = 1, p<0.001). Red snapper shrimp consumption also approached a higher asymptotic level than did vermilion snapper shrimp consumption.

## Discussion

Red snapper and vermilion snapper show spatial overlap in the northern GOM between Mobile Bay and Saint Andrew’s Bay and rely heavily upon similar prey resources; thus, dietary overlap between these two species is probable. Red and vermilion snapper were the dominant reef predators across the study area both in terms of abundance and influence on the reef fish assemblage. While the two species appear to have differing habitat preferences, red and vermilion snapper cohabited the overwhelming majority of reefs sampled. Likewise, red and vermilion snapper rely heavily upon similar prey resources, although the magnitude of consumption across some prey categories differed. Red and vermilion snapper share four of their five most important prey categories according to %N and %W, and three of their five most important categories by %IRI. Our findings add to a body of literature documenting feeding preferences for these two species. Whereas we show a significant effect of habitat (natural vs. artificial) on the diet of red snapper, previous studies have documented little difference in the diet of red snapper sampled on natural vs. artificial reefs [13, 19, 20]. This may be due to geographical differences in reef types, for example pyramids vs. toppled rigs, or hard rock vs sand and soft corals, across the northern GOM, as suggested by others [20]. Our findings demonstrate seasonal differences in vermilion snapper consumption of zooplankton, similar to seasonal shifts in zooplankton consumption shown for red snapper [11]. Cumulatively, these findings illustrate the importance of examining the feeding ecology of these two co-occurring species concurrently, and across the range of habitats they occupy.

Dietary overlap was significant in this study, despite large size discrepancies between the red snapper and vermilion snapper sampled. The primary difference between these two species’ diets is the relative importance of fish and amphipods. Yet, by all measures each category ranks in the top four most important categories for both species. Wells et al. [19] indicated that red snapper smaller than 400 mm FL, which were underrepresented in this study, feed less upon fish and consume a larger proportion of small invertebrates including amphipods. Consequently, dietary overlap may be higher between small red snapper and vermilion snapper. Conversely, McCawley and Cowan [11] and Tarnecki and Patterson [13] both found that plankton consumption increased as red snapper became larger. Tarnecki and Patterson [13] attributed the increase in plankton consumption to the red snapper’s opportunistic feeding strategy, because plankton consumption did not differ between habitat types. McCawley and Cowan [11] hypothesized that the high density of artificial reefs could force red snapper to rely on alternative prey resources to meet bioenergetic demands. Our results corroborate this hypothesis, as red snapper relied more upon lower trophic-level prey such as amphipods and tunicates at artificial reefs than at natural reefs. Amphipods and tunicates were nearly absent from red snapper stomach contents collected from natural reefs, and %W of fish more than doubled that for stomachs collected from artificial reefs. If large red snapper increase their consumption of lower trophic-level prey such as amphipods in response to high conspecific density in artificial reef zones then they may adversely affect vermilion snapper and other species relying heavily on those same prey resources.

Dietary overlap between red snapper and vermilion snapper was high at single species sites and lower at sites they cohabited, a possible indicator of competition and resource partitioning. The PERMANOVA indicated that the effects of cohabitation on dietary overlap are independent of reef type and depth. If dietary overlap exists, then based on the competitive exclusion principle [21], resource partitioning is expected. The discrepancies between red and vermilion snapper’s reliance upon fish and amphipods were exacerbated at sites the species cohabited. Vermilion snapper consumed 39% less fish and more than twice as many amphipods by weight at sites where red snapper co-occurred. Resource partitioning in the prey categories of amphipods and fish may allow for spatial overlap between red and vermilion snapper.

Findings from the manipulative mesocosm experiments demonstrated that red and vermilion snapper of similar sizes displayed significant differences in feeding behavior. Red snapper fed at a higher rate and consumed more shrimp per fish than vermilion snapper. However, interspecific competition with red snapper did not have a significant effect upon vermilion snapper prey consumption compared to conspecific competition in the single species treatment. So, red snapper did not competitively exclude vermilion snapper from the prey resource in the experiment. However, this result may have been due to the experimental design. Both species were allowed to approach satiation, so prey was effectively not a limited resource in the time period of the trials. Consequently, the experiment only tested for the effects of interference competition and did not test for the effects of exploitative competition. In the situation of limited prey, it is expected that red snapper would exclude vermilion snapper from prey as they feed more successfully and at a higher rate.

Red snapper prey consumption was higher when red snapper and vermilion snapper foraged together in the mixed species treatment; however, this is likely not the result of facilitation between two predators. While not common [22], facilitation between predators in a marine system has previously been documented. However, no mechanism of facilitation between the competitors was apparent in this study. Rather, vermilion snapper’s lower feeding rate made more prey available to red snapper in the mixed species treatment in comparison to the single species treatment. Video analysis was essential to gain this insight. Manipulative experiments concerning multiple predator interactions have traditionally been limited to recapturing and counting prey (e.g. [23]). Video analysis allows the researcher to noninvasively determine the prey consumed by each predator species. Furthermore, video allows for more detailed analysis such as feeding rate and success rate, since individual predation events may be recorded. Given the affordability of unobtrusive high definition camera equipment, we suggest further use of video analysis in manipulative experiments.

While the presented spatial and dietary overlap data suggests the possibility of competition between red and vermilion snapper and warrants further exploration, the methods and results presented do not allow us to definitively conclude that competition between red and vermilion snapper exists. Both the spatial and dietary overlap analyses are limited in resolution, and have methodological limitations. Despite the obvious benefits of a non-extractive sampling method like ROV, these visual survey data are not without bias. Synthesis across 48 marine taxa suggests that attraction to or avoidance of a ROV is a common bias [24]. This bias may vary by species, for example between the red and vermilion snapper in the current study; however, quantifying such variation would require future experimental manipulations. Similarly, it is possible that red and vermilion snapper partition resources at finer resolutions than explored in this study [25, 26]. Further research into the interactions between these two species will involve an ongoing debate regarding red snapper feeding ecology. While some studies have contended that red snapper feed heavily upon reef-associated prey [27], others have speculated that red snapper use reefs primarily for shelter and rove the surrounding seafloor to feed [11, 20]. If the latter is correct, or even if red snapper employ both strategies, an interesting scenario can be envisioned. Red snapper may feed opportunistically upon higher quality prey close to the reef site, but once depleted they may leave the reef to seek more prey. Vermilion snapper may also feed opportunistically upon the same prey at the reef site but may not exploit those resources as successfully as red snapper. Rather than leave the reef in search of more prey, under this hypothesis vermilion snapper, which are thought to have high site fidelity [28, 29, 30] may stay close to the reef and feed at a lower rate or upon less desirable prey. Johnson et al [12] proposed a similar idea, as they found that vermilion snapper in the northern GOM fed heavily upon normally undesirable benthic tunicates. These behaviors would also be consistent with differences in growth, as red snapper off Alabama have been shown to have faster growth than vermilion snapper of a similar size [12, 31]. Predatory fish with high growth rates, such as tunas and larger mackerels, tend to roam constantly in search of high concentrations of high quality food. According to this hypothesis red snapper would employ this strategy, although to a lesser degree. Future studies using high-resolution telemetry to examine fine-scale movement of red snapper relative to reefs (e.g. [32]) are needed to further examine these alternate hypotheses.

The methods used to analyze red and vermilion snapper diets limited the resolution and certainty of the results. The issues associated with gut content analysis are well documented [16]. A very large proportion of stomachs contents was unidentifiable chyme. Furthermore, much of the identifiable gut contents could only be identified to broad levels (e.g. “Unid. Bony Fish”, “Amphipods”). As such, it is possible that red and vermilion snapper partition prey resources at a finer resolution than presented in this paper. While stable isotope analysis eliminates some of the biases associated with stomach content analysis [16], this popular method could not resolve fine scale differences between red and vermilion snapper diets. In the future, technologically advanced methods such as DNA barcoding could allow for very high resolution in stomach content analysis and could allow for identification of visually unidentifiable contents.

Previous reviews of reef fish competition [26] have suggested that manipulative experiments in the field are the best method for determining the existence of competition. Connell [33] described four basic categories of evidence for interspecific competition and argued they increased in strength of inference in the following order: 1) comparison of resource use and subsequent inference of resource partitioning 2), comparison of resource use in sympatric vs. allopatric conditions, which Connell termed “natural experiments”, 3) observation of unambiguous interference competition (e.g. territoriality), and 4) manipulative experiments in the field. We have provided evidence falling into the first two categories that cumulatively suggests potential for exploitative competition between red and vermilion snapper. The results from the mesocosm experiment indicate that interference competition is unlikely under non-impaired conditions [34]. However, either species could become subordinate if out-numbered or out-sized by the other, so further experimentation under impaired conditions is necessary to rule out interference competition [34]. We cannot conclusively determine the existence of competition between red and vermilion snapper without manipulative field experiments [26, 33]; however, the overlap data and evidence of resource partitioning presented herein warrants such further experimentation. Lindberg et al. [35] showed that gag select habitats that offer superior shelter at the expense of conspecific competition for prey resources limiting their growth. If red or vermilion snapper behave similarly, they may continue to cohabit and even favorably recruit to a reef even if interspecific competition inhibits that species’ growth. Thus, manipulative experiments should focus on measures of condition such as growth rates and fecundity in addition to abundance as indicators of the effects of interspecific competition. Furthermore, effects on growth rates and fecundity are especially relevant to fisheries management, as fisheries managers aim to maximize productivity of a stock.

Fishing pressure has fundamentally altered the northern Gulf of Mexico fisheries ecosystem [36]. Through field sampling and manipulative experimentation, our findings demonstrate that there is significant potential for competition between two commercially valuable components of the Gulf of Mexico ecosystem, red snapper and vermilion snapper, as these two species overlap both spatially and trophically. Furthering our understanding of the nature of the interactions between these two species will require research on how these two species partition space and prey resources at a finer scale. Additionally, manipulative experiments in the field may be required to conclusively determine the existence of competition and understand the effects of competition on each species’ welfare. Our results highlight the importance of examining the potential for interactions between co-occurring species in the Gulf of Mexico reef fish complex.

## Supporting Information

S1 Fig GoPro tank placement. Schematic diagram of GoPro placement in tanks.
(TIF)

S1 File Feeding attempts. Examples of failed and successful feeding attempts and red and vermilion snapper.
(MOV)

S1 Table Detailed diet data. Complete list (n = 46) of unique prey taxa identified from stomachs of red and vermilion snapper.
(DOCX)
