# Supplementary figures and images for: Spatial and Dietary Overlap Creates Potential for Competition between Red Snapper (Lutjanus campechanus) and Vermilion snapper (Rhomboplites aurorubens)
Source: PLoS One. 2015 Dec 2;10(12):e0144051. doi: 10.1371/journal.pone.0144051 (PMC4667897; doi:10.1371/journal.pone.0144051)

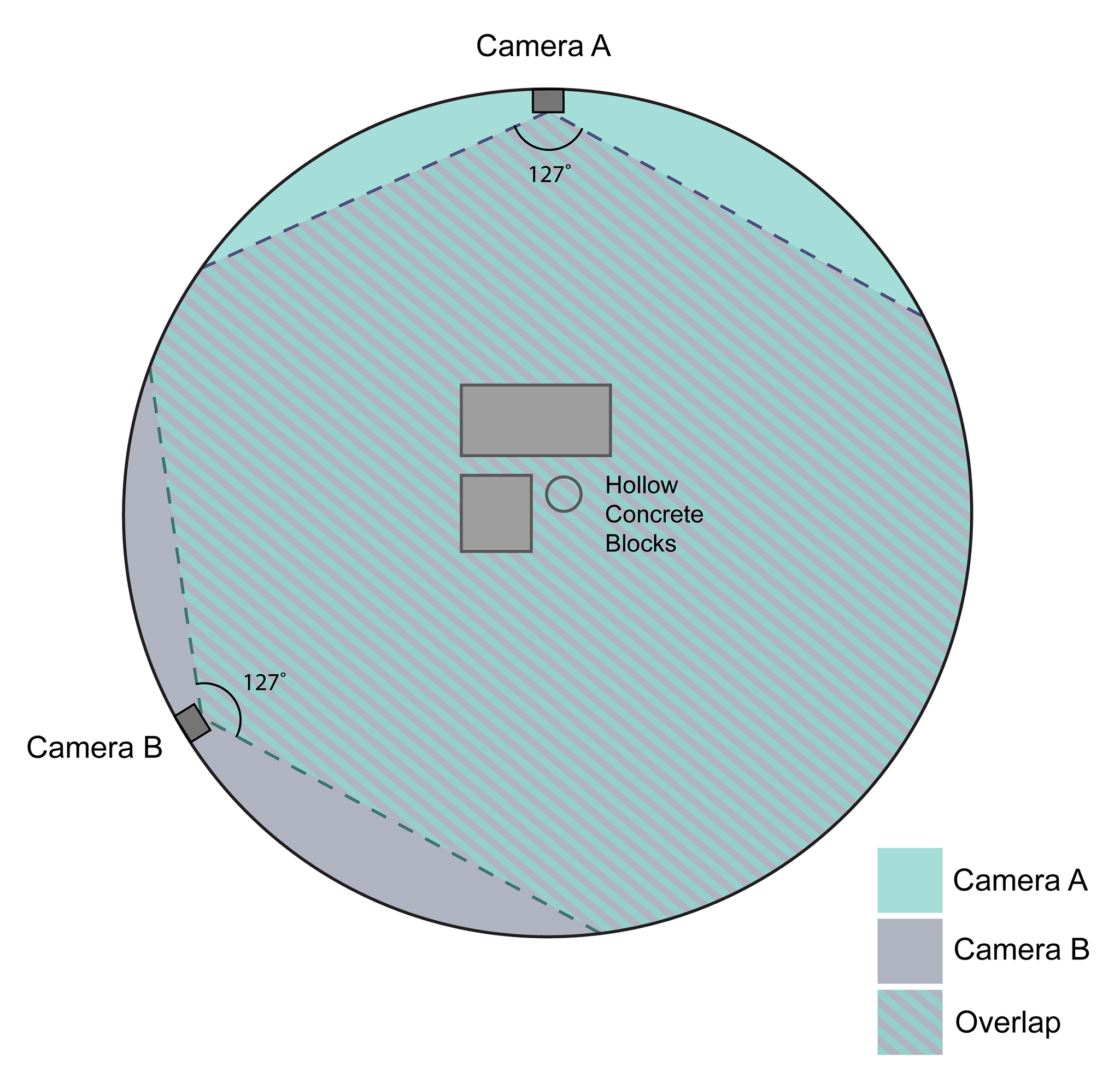

Supplement: S1 Fig — Schematic diagram of GoPro placement in tanks. (TIF) [file pone.0144051.s001.tif]
