# Supplementary material for: Spatial and Dietary Overlap Creates Potential for Competition between Red Snapper (Lutjanus campechanus) and Vermilion snapper (Rhomboplites aurorubens)
Source: PLoS One. 2015 Dec 2;10(12):e0144051. doi: 10.1371/journal.pone.0144051 (PMC4667897; doi:10.1371/journal.pone.0144051)
Supplement: S1 Table — Complete list (n = 46) of unique prey taxa identified from stomachs of red and vermilion snapper. (DOCX) [file pone.0144051.s003.docx]

|  |  |  | Red Snapper | | | | | |  | Vermilion Snapper | | | | | |
| --- | --- | --- | --- | --- | --- | --- | --- | --- | --- | --- | --- | --- | --- | --- | --- |
|  |  |  | %N | | %W | | %O | %IRI |  | %N | | %W | | %O | %IRI |
| Prey Category | |  | Mean | SE | Mean | SE | Value | Value |  | Mean | SE | Mean | SE | Value | Value |
| Amphipoda | |  |  |  |  |  |  |  |  |  |  |  |  |  |  |
|  | Phronima |  | 0.07 | 0.07 | 0.27 | 0.27 | 0.76 | 0.01 |  | 0.68 | 0.65 | 1.12 | 1.12 | 2.17 | 0.08 |
|  | Unidentified |  | 11.16 | 2.45 | 7.92 | 2.19 | 16.67 | 10.21 |  | 24.90 | 3.49 | 21.00 | 3.72 | 48.91 | 45.31 |
| Barnacle | |  | 0.76 | 0.76 | 0.79 | 0.79 | 0.76 | 0.04 |  | 0.00 | 0.00 | 0.00 | 0.00 | 0.00 | 0.00 |
| Bivalve | |  | 0.14 | 0.11 | 0.07 | 0.05 | 1.52 | 0.01 |  | 0.00 | 0.00 | 0.00 | 0.00 | 0.00 | 0.00 |
| Chaetognath | |  | 0.03 | 0.03 | 0.06 | 0.06 | 0.76 | 0.00 |  | 0.00 | 0.00 | 0.00 | 0.00 | 0.00 | 0.00 |
| Copepod | |  |  |  |  |  |  |  |  |  |  |  |  |  |  |
|  | Calanoid |  | 4.66 | 1.55 | 0.10 | 0.07 | 8.33 | 1.27 |  | 9.03 | 2.39 | 5.44 | 2.15 | 23.91 | 6.98 |
|  | Cyclopoid |  | 1.24 | 0.65 | 0.06 | 0.06 | 6.06 | 0.25 |  | 1.72 | 0.82 | 0.12 | 0.08 | 7.61 | 0.28 |
| Coral | |  | 0.76 | 0.76 | 0.79 | 0.79 | 0.76 | 0.04 |  | 0.00 | 0.00 | 0.00 | 0.00 | 0.00 | 0.00 |
| Crabs | |  |  |  |  |  |  |  |  |  |  |  |  |  |  |
|  | Majjid |  | 0.25 | 0.25 | 0.33 | 0.33 | 0.76 | 0.01 |  | 0.00 | 0.00 | 0.00 | 0.00 | 0.00 | 0.00 |
|  | Portunid |  | 3.33 | 1.29 | 3.74 | 1.51 | 6.82 | 1.55 |  | 0.07 | 0.07 | 1.11 | 1.11 | 1.09 | 0.03 |
|  | Calappidae |  | 1.52 | 0.92 | 1.73 | 1.12 | 2.27 | 0.24 |  | 0.00 | 0.00 | 0.00 | 0.00 | 0.00 | 0.00 |
|  | Dromiidae |  | 0.00 | 0.00 | 0.00 | 0.00 | 0.00 | 0.00 |  | 0.22 | 0.22 | 0.00 | 0.00 | 1.09 | 0.00 |
|  | Unidentified |  | 4.55 | 1.57 | 5.61 | 1.79 | 12.12 | 3.96 |  | 11.71 | 2.95 | 11.66 | 3.01 | 26.09 | 12.30 |
| Fish | |  |  |  |  |  |  |  |  |  |  |  |  |  |  |
|  | Lizardfish |  | 0.47 | 0.39 | 0.90 | 0.70 | 1.52 | 0.07 |  | 0.00 | 0.00 | 0.00 | 0.00 | 0.00 | 0.00 |
|  | Pipefish |  | 0.76 | 0.76 | 0.79 | 0.79 | 0.76 | 0.04 |  | 0.00 | 0.00 | 0.00 | 0.00 | 0.00 | 0.00 |
|  | Lancer Stargazer | | 0.06 | 0.06 | 0.79 | 0.79 | 0.76 | 0.02 |  | 0.00 | 0.00 | 0.00 | 0.00 | 0.00 | 0.00 |
|  | Gulf Butterfish | | 0.76 | 0.76 | 0.79 | 0.79 | 0.76 | 0.04 |  | 0.00 | 0.00 | 0.00 | 0.00 | 0.00 | 0.00 |
|  | Atlantic Chub Mackerel | | 3.29 | 1.47 | 3.31 | 1.48 | 3.79 | 0.80 |  | 0.00 | 0.00 | 0.00 | 0.00 | 0.00 | 0.00 |
|  | Balistidae |  | 0.08 | 0.08 | 0.76 | 0.76 | 0.76 | 0.02 |  | 0.00 | 0.00 | 0.00 | 0.00 | 0.00 | 0.00 |
|  | Scad spp. |  | 0.38 | 0.27 | 0.47 | 0.37 | 1.52 | 0.04 |  | 0.00 | 0.00 | 0.00 | 0.00 | 0.00 | 0.00 |
|  | Gulf Menhaden | | 0.30 | 0.30 | 0.55 | 0.55 | 0.76 | 0.02 |  | 0.00 | 0.00 | 0.00 | 0.00 | 0.00 | 0.00 |
|  | Eel |  | 1.84 | 1.10 | 2.50 | 1.30 | 3.03 | 0.42 |  | 0.00 | 0.00 | 0.00 | 0.00 | 0.00 | 0.00 |
|  | Unidentified |  | 19.94 | 3.00 | 25.36 | 3.56 | 35.61 | 51.77 |  | 10.93 | 2.79 | 16.66 | 3.74 | 25.00 | 13.92 |
| Fish Lice | |  | 0.63 | 0.45 | 0.13 | 0.13 | 1.52 | 0.04 |  | 0.00 | 0.00 | 0.00 | 0.00 | 0.00 | 0.00 |
| Galitheidae | |  | 0.09 | 0.09 | 0.02 | 0.02 | 0.76 | 0.00 |  | 0.36 | 0.36 | 0.05 | 0.05 | 1.09 | 0.01 |
| Gastropod | |  |  |  |  |  |  |  |  |  |  |  |  |  |  |
|  | Pteropod |  | 13.88 | 2.79 | 12.97 | 2.70 | 18.94 | 16.32 |  | 2.42 | 1.32 | 1.83 | 1.23 | 7.61 | 0.65 |
|  | Moon Snail |  | 0.38 | 0.38 | 0.78 | 0.78 | 0.76 | 0.03 |  | 0.00 | 0.00 | 0.00 | 0.00 | 0.00 | 0.00 |
|  | Unidentified |  | 2.59 | 0.99 | 2.26 | 1.08 | 6.82 | 1.06 |  | 2.01 | 1.22 | 1.30 | 1.13 | 7.61 | 0.51 |
| Hermit Crab | |  | 0.00 | 0.00 | 0.00 | 0.00 | 0.00 | 0.00 |  | 0.33 | 0.18 | 0.40 | 0.22 | 7.61 | 0.11 |
| Isopod | |  | 0.09 | 0.09 | 0.03 | 0.03 | 0.76 | 0.00 |  | 0.49 | 0.47 | 1.12 | 1.12 | 2.17 | 0.07 |
| Krill | |  | 0.28 | 0.28 | 0.31 | 0.31 | 0.76 | 0.01 |  | 3.67 | 1.57 | 2.68 | 1.40 | 11.96 | 1.53 |
| Luciferidae | |  | 0.00 | 0.00 | 0.00 | 0.00 | 0.00 | 0.00 |  | 0.36 | 0.36 | 0.00 | 0.00 | 1.09 | 0.01 |
| Mantis Shrimp | |  | 4.50 | 1.57 | 5.69 | 1.85 | 12.12 | 3.96 |  | 7.03 | 2.02 | 12.05 | 3.08 | 23.91 | 9.21 |
| Mole Crab | |  | 1.52 | 1.07 | 1.57 | 1.11 | 1.52 | 0.15 |  | 0.00 | 0.00 | 0.00 | 0.00 | 0.00 | 0.00 |
| Mysida | |  | 0.25 | 0.25 | 0.04 | 0.04 | 0.76 | 0.01 |  | 0.64 | 0.46 | 0.72 | 0.57 | 4.35 | 0.12 |
| Ostracod | |  | 1.84 | 1.00 | 0.81 | 0.79 | 4.55 | 0.39 |  | 3.60 | 1.51 | 1.13 | 0.72 | 14.13 | 1.35 |
| Phyllosoma | |  | 2.32 | 1.11 | 2.83 | 1.35 | 4.55 | 0.75 |  | 1.04 | 0.56 | 1.68 | 1.05 | 6.52 | 0.36 |
| Polychaete | |  | 1.99 | 1.10 | 2.01 | 1.14 | 3.79 | 0.49 |  | 2.08 | 1.20 | 3.36 | 1.84 | 6.52 | 0.72 |
| Shrimp | |  |  |  |  |  |  |  |  |  |  |  |  |  |  |
|  | Caridean Shrimp | | 0.04 | 0.04 | 0.08 | 0.08 | 0.76 | 0.00 |  | 0.00 | 0.00 | 0.00 | 0.00 | 0.00 | 0.00 |
|  | Penaeid Shrimp | | 0.08 | 0.08 | 0.13 | 0.13 | 0.76 | 0.01 |  | 1.38 | 1.11 | 1.32 | 1.14 | 3.26 | 0.18 |
|  | Rock Shrimp |  | 0.76 | 0.76 | 0.79 | 0.79 | 0.76 | 0.04 |  | 0.00 | 0.00 | 0.00 | 0.00 | 0.00 | 0.00 |
|  | Unidentified |  | 4.00 | 1.33 | 4.28 | 1.60 | 11.36 | 3.02 |  | 5.39 | 1.79 | 5.82 | 1.98 | 16.30 | 3.69 |
| Squid | |  | 3.83 | 1.51 | 3.48 | 1.56 | 6.06 | 1.42 |  | 0.40 | 0.36 | 1.11 | 1.09 | 3.26 | 0.10 |
| Tunicate | |  |  |  |  |  |  |  |  |  |  |  |  |  |  |
|  | Salp |  | 2.95 | 1.20 | 3.31 | 1.35 | 6.82 | 1.37 |  | 4.78 | 2.04 | 6.67 | 2.51 | 6.52 | 1.51 |
|  | Unidentified |  | 1.01 | 0.80 | 0.79 | 0.79 | 1.52 | 0.09 |  | 0.00 | 0.00 | 0.00 | 0.00 | 0.00 | 0.00 |
| Unidentified Egg | |  | 0.64 | 0.64 | 0.00 | 0.00 | 0.76 | 0.02 |  | 4.75 | 2.00 | 1.63 | 1.17 | 7.61 | 0.98 |
